# Disordered Crystal Structure and Anomalously High Solubility of Radium Carbonate

**DOI:** 10.1021/acs.inorgchem.3c01513

**Published:** 2023-07-21

**Authors:** Artem V. Matyskin, Burçak Ebin, Stefan Allard, Natallia Torapava, Lars Eriksson, Ingmar Persson, Paul L. Brown, Christian Ekberg

**Affiliations:** † Nuclear Chemistry and Industrial Materials Recycling Group, Energy and Materials Division, Department of Chemistry and Chemical Engineering, Chalmers University of Technology, Kemivägen 4, SE-41296 Gothenburg, Sweden; ‡ MAX IV Laboratory, Lund University, Fotongatan 2, SE-22594 Lund, Sweden; § Arrhenius Laboratory, Department of Materials and Environmental Chemistry, Stockholm University, Svante Arrhenius väg 16 C, SE-11691 Stockholm, Sweden; ∥ Department of Molecular Sciences, Swedish University of Agricultural Sciences, P.O. Box 7015, SE-75007 Uppsala, Sweden; ⊥ Rio Tinto Development and Technology, 1 Research Avenue, 3083 Bundoora, Victoria, Australia

## Abstract

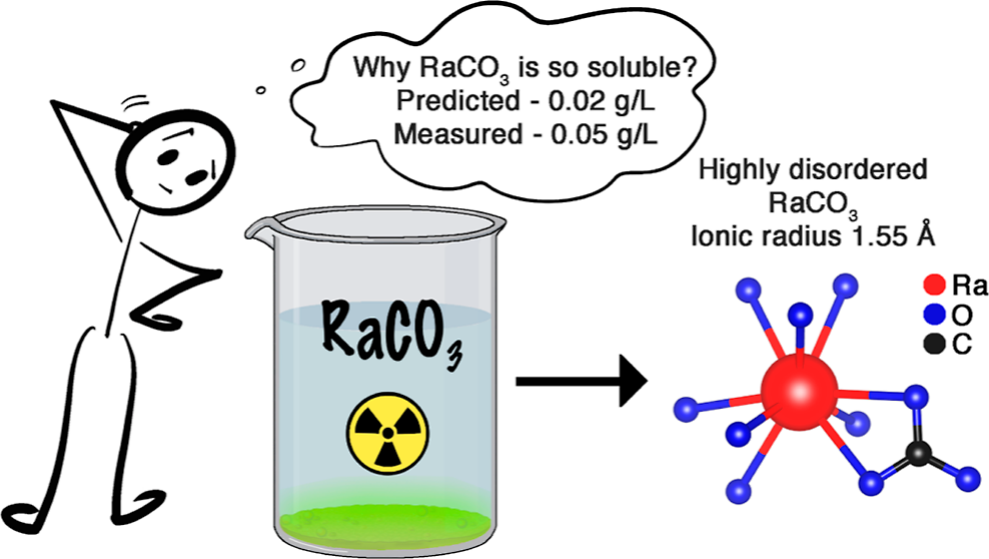

Radium-226 carbonate was synthesized from radium–barium sulfate (^226^Ra_0.76_Ba_0.24_SO_4_) at room temperature and characterized by X-ray powder diffraction (XRPD) and extended X-ray absorption fine structure (EXAFS) techniques. XRPD revealed that fractional crystallization occurred and that two phases were formed—the major Ra-rich phase, Ra(Ba)CO_3_, and a minor Ba-rich phase, Ba(Ra)CO_3_, crystallizing in the orthorhombic space group *Pnma* (no. 62) that is isostructural with witherite (BaCO_3_) but with slightly larger unit cell dimensions. Direct-space *ab initio* modeling shows that the carbonate oxygens in the major Ra(Ba)CO_3_ phase are highly disordered. The solubility of the synthesized major Ra(Ba)CO_3_ phase was studied from under- and oversaturation at 25.1 °C as a function of ionic strength using NaCl as the supporting electrolyte. It was found that the decimal logarithm of the solubility product of Ra(Ba)CO_3_ at zero ionic strength (log_10_*K*_sp_^0^) is −7.5(1) (2σ) (*s* = 0.05 g·L^–1^). This is significantly higher than the log_10_*K*_sp_^0^ of witherite of −8.56 (*s* = 0.01 g·L^–1^), supporting the disordered nature of the major Ra(Ba)CO_3_ phase. The limited co-precipitation of Ra^2+^ within witherite, the significantly higher solubility of pure RaCO_3_ compared to witherite, and thermodynamic modeling show that the results obtained in this work for the major Ra(Ba)CO_3_ phase are also applicable to pure RaCO_3_. The refinement of the EXAFS data reveals that radium is coordinated by nine oxygens in a broad bond distance distribution with a mean Ra–O bond distance of 2.885(3) Å (1σ). The Ra–O bond distance gives an ionic radius of Ra^2+^ in a 9-fold coordination of 1.545(6) Å (1σ).

## Abstract 2

XRD measurements of RaCO_3_ revealed that it is not isostructural with witherite, and direct-space *ab initio* modeling showed that the carbonate oxygens are highly disordered. It was found that the solubility of RaCO_3_ is unexpectedly higher than the solubility of witherite (log_10_*K*_sp_^0^ = −7.5 and −8.56, respectively), supporting the disordered nature of RaCO_3_. EXAFS data revealed an ionic radius of Ra^2+^ of 1.55 Å. Radium is the only alkaline-earth metal which forms disordered crystals in its carbonate phase.

## 1 Introduction

Radium is the heaviest alkaline-earth metal and has no stable isotopes. It occurs in the earth’s crust in only trace quantities (≈1 pg·g^–1^)^1^ as part of the ^238^U, ^235^U, and ^232^Th radioactive decay series. The most long-lived and abundant radium isotope is ^226^Ra with a half-life of 1600 years. Radium is among the most radiotoxic elements,^2^ and if ingested, it follows similar pathways to calcium and concentrates mostly in bones and bone marrow and can cause bone sarcoma.^3^ The decay chain of ^226^Ra includes many alpha-, beta-, and gamma-emitting short-lived radionuclides, and dose rates even from milligram quantities of radium are significant. Moreover, ^226^Ra decays to the radioactive noble gas radon, ^222^Rn (*t*_1/2_ = 3.82 days), which is also an alpha-emitter. Handling of volatile alpha emitters requires rigorous safety precautions, and as a result, experimental work even with small quantities of radium compounds is challenging, as is the case for other highly radioactive elements.^4,5^

The high radiotoxicity of radium and its decay products requires an understanding of its migration in the environment from technologically enhanced naturally occurring radioactive materials. Moreover, in 2013, a ^223^Ra^2+^ (*t*_1/2_ = 11.43 days) saline solution (trademark Xofigo^©^) was approved by the U.S. Food and Drug Administration and later by the European Medicines Agency for treatment of patients with castration-resistant prostate carcinomas, symptomatic bone metastases, and no known visceral metastatic disease. To date, ^223^Ra^2+^ saline solution is the first and only approved radiopharmaceutical for targeted alpha therapy, but other radiopharmaceuticals with radium, for example, CaCO_3_ microparticles radiolabeled with ^224^Ra (*t*_1/2_ = 3.63 days), are under development for local therapy of disseminated cancers and are undergoing clinical trials.^6,7^

Knowledge of the fundamental chemical properties of radium is required to understand its behavior in the environment and to exploit the therapeutic potential of radium in metastatic disease treatment. However, its chemistry remains unexplored compared with the other non-radioactive alkaline-earth metals due to the extreme rarity of radium as a laboratory material and its highly radioactive nature.^8^ For example, the paper by Shannon^9^ is considered one of the most comprehensive and accurate set of ionic radii; however, it includes ionic radii for radium only in 8- and 12-fold coordination but not in 9-fold. Moreover, all radium radii were estimated by Shannon^9^ from plots of ionic radii (*r*^3^) against unit cell volume (*V*) of isostructural series assuming that the relationship between *r*^3^ and *V* is linear. To the best of our knowledge, there are only two studies, where interatomic distances in radium compounds have been measured experimentally: Hedström and co-workers^10^ studied solid radium–barium sulfate using extended X-ray absorption fine structure (EXAFS) and recently Yamaguchi et al.^11^ measured diluted HNO_3_ with 2 MBq of ^226^Ra (≈4 mM) also using EXAFS. Therefore, experimental studies of radium will extend the knowledge of alkaline-earth metal chemistry applicable on both fundamental and applied levels.

Generally, it is assumed that radium chemistry is similar to that of barium due to their similar chemical and physical properties.^8^ For example, radium migration in natural waters is mainly controlled by its co-precipitation with other alkaline-earth metal sulfate minerals, mostly with barite (BaSO_4_). However, radium mobility in some natural sulfate-free waters can be controlled by its co-precipitation with various carbonate minerals, moslty with witherite (BaCO_3_).^12^ The mechanism and degree of radium co-precipitation with witherite depends mainly on crystal structures and the solubilities of pure witherite and RaCO_3_. The crystal structure and solubility of witherite and other non-radioactive alkaline-earth metal carbonates are well established, but little is known about the properties of RaCO_3_. Only two papers deal with the crystal structure of RaCO_3_. It was studied experimentally by Weigel and Trinkl^13^ in 1973 and by Butkalyuk and co-workers^14^ in 2013. In both papers, the RaCO_3_ samples studied were obtained by calcination (3.5 h at 640 °C and 8 h at 800 °C, respectively) and characterized using the X-ray powder diffraction (XRPD) technique. The XRPD patterns obtained are in good agreement and reveal that calcined RaCO_3_ is isostructural with strontianite (SrCO_3_), witherite, and cerussite (PbCO_3_), crystallizing in the space group *Pnma* (no. 62).

To the best of our knowledge, an experimentally determined RaCO_3_ solubility product has never been reported in the literature, although an indication that the solubility of RaCO_3_ is significantly higher than that of witherite was first given by Nikitin^15^ in 1937. Nikitin performed the following experiment: 100 mL of a solution containing 2 g of (NH_4_)_2_CO_3_, 5 g of NH_4_Cl, and 10 mL of concentrated NaOH was added to 40 mL of a pure concentrated radium solution. The formed precipitate was filtered and a mixture of HCl and H_2_SO_4_ was added to the filtrate to precipitate RaSO_4_. Then, the mass of the precipitated RaSO_4_ was measured. Nikitin did the same experiment with pure barium and found that the mass of precipitated RaSO_4_ was approximately 10 times higher than the mass of precipitated BaSO_4_, which means that the solubility product of RaCO_3_ is approximately 10 times larger than that of BaCO_3_ under the experimental conditions used. At that time, the theories for calculation of activity coefficients in such concentrated solutions (Specific ion Interaction Theory or Pitzer formalism) were not developed yet. Nowadays, there is evidence that Ra^2+^ and Ba^2+^ have similar activity coefficients in chloride and hydroxide media.^16,17^ Therefore, it can be argued that the same difference (10 times) in solubilities of BaCO_3_ and RaCO_3_ can be expected at zero ionic strength. Moreover, recently Brown and co-workers^18^ used thermodynamic modeling to derive the solubility of pure radium carbonate and showed that the decimal logarithm of the solubility product of pure radium carbonate at zero ionic strength and 25 °C is log_10_*K*_sp_^0^ = −7.57 (*s* = 0.047 g·L^–1^). This value is in good agreement with the experimental results obtained by Nikitin and is significantly different from the solubility product of witherite at zero ionic strength (log_10_*K*_sp_^0^ = −8.56).^19^ This may indicate that non-calcined RaCO_3_ is not isostructural with witherite.

Goldschmidt^20^ was the first to show that radium can co-precipitate with strontianite, witherite, and cerussite in aqueous solution and determined its partition coefficients in these phases. Co-precipitation of trace radium with witherite, aragonite, calcite, and other minerals has been recently studied by other researchers^21−25^ and has also been reviewed.^26,27^ All reported partition (crystallization) coefficients of radium in witherite are below unity, which means that most of the radium stays in the aqueous phase and that pure RaCO_3_ is more soluble than pure witherite. The limited co-precipitation of radium with aragonite and witherite also indicates that RaCO_3_ may have a different crystal structure than these minerals.

However, the commonly accepted solubility product of RaCO_3_ in the literature is from Langmuir and Riese.^12^ They assumed that RaCO_3_ is isostructural with witherite and plotted logarithms of the solubility products of strontianite, witherite, and RaCO_3_ as functions of the effective ionic radii of Sr^2+^, Ba^2+^, and Ra^2+^ in 8-fold coordination and estimated a log_10_*K*_sp_^0^ for RaCO_3_ of −8.3 (*s* = 0.02 g·L^–1^) at ambient conditions and zero ionic strength.

The present study attempted to refine the crystal structure parameters of radium carbonate and gain a better understanding of the radium co-precipitation mechanism with witherite and other carbonate phases. For this purpose, a radium–barium carbonate co-precipitate was synthesized, characterized by XRPD and EXAFS techniques, and modeled using direct space methods. The solubility of RaCO_3_ was experimentally studied as a function of ionic strength at 25.1 °C, and the apparent solubility products of RaCO_3_ determined were extrapolated to zero ionic strength to obtain the thermodynamic value for the solubility product of RaCO_3_.

## 2 Methods

**Warning**: Radium sources used in this work are highly radioactive and emit the short-lived α-emitting gas—radon. The experimental work described requires rigorous safety precautions including working in radiological fume hoods, gloveboxes, and hot cells equipped with a Rn capture system.

### 2.1 Chemicals

All experimental work with radium powder samples was performed in gloveboxes or hot cells at negative pressure and equipped with a radon capture system to avoid contamination, inhalation of radon, and ingestion of radium. All solutions were prepared using an analytical balance (Sartorius Quintix125D-1S). All chemicals used in this work are listed in Table 1.

**Table 1:** Chemicals Used^a^

chemical	source	purity
^226^Ra(Ba)CO_3_ powder	synthesized in house from radium sulfate^22^	mole fractions: Ra = 0.756 ± 0.010, Ba = 0.244 ± 0.006, Pb = 0.002^28^
^226^Ra stock solution in 2.77 mol·L^–1^ HCl	synthesized in house from radium sulfate^22^ using HCl from Merck (Suprapur)	mole fractions: Ra = 0.756 ± 0.005, Ba = 0.244 ± 0.003, Pb = 0.002^28^
BaSO_4_	Sigma-Aldrich	99.998% trace metals basis
Na_2_CO_3_	Sigma-Aldrich	99.999% trace metals basis
NaOH	Sigma-Aldrich (Fluka) standard solution	99.9%
Na_2_EDTA·2H_2_O	Sigma-Aldrich	99% molecular biology grade
HCl	Sigma-Aldrich	99.999% trace metals basis
H_2_O	Type 1 Merck Milli-Q	18.2 MΩ·cm at 25 °C, total organic content <5 mg·L^–1^
ethanol	Solveco	Aa grade, 99.7%

a Uncertainties of Ra and Ba mole fractions are 2σ standard deviations.

### 2.2 Synthesis of Radium Carbonate

The initial source of radium for this work was used for brachytherapy in the 1940s to 1960s and was in the form of a steel flat plaque with five platinum-gold cylinders sealed inside the plaque. Each cylinder contained 20 mg of radium–barium sulfate powder. Disassembly of the radium source and synthesis of the radium–barium carbonate from radium–barium sulfate powder was performed as previously described^22^—the radium–barium sulfate powder was heated in 1.5 mol·L^–1^ aqueous solution of Na_2_CO_3_ up to ca. 85 °C using a heating mantle. After about 90 min of heating, the solution was cooled, and the supernatant was removed. The procedure was repeated twice more. Pure BaCO_3_ was synthesized from BaSO_4_ using the same procedure and chemicals as used for the radium–barium carbonate synthesis. Small portions of the initial radium–barium sulfate and synthesized radium–barium carbonate were dissolved in 0.1 mol·L^–1^ Na_2_EDTA solution and in 0.1 mol·L^–1^ hydrochloric acid solution, respectively, and their purities were measured using a sector field inductively coupled plasma mass spectrometer.^28^

### 2.3 XRPD and EXAFS Data Collection

The sample for the XRPD study was prepared using a similar procedure to that previously described.^28^ The whole batch of synthesized radium–barium carbonate powder was heated for 4 h at 200–250 °C using a heating mantel. After cooling, approximately 0.5 mg of radium–barium carbonate powder was placed on a low background silicon air-tight sample holder, and several drops of ethanol were added to evenly distribute the powder on the sample holder. After ethanol evaporation, the sample holder with the radium–barium carbonate powder was closed using a screw dome and transferred to another glovebox with the X-ray powder diffractometer inside. The sample holder was then placed in the measuring position, fixed in place, and the dome was removed.

The XRPD measurements of radium–barium carbonate and pure synthesized BaCO_3_ were collected using the same procedure as described previously.^28^ Both samples were approximately of the same size and were measured at 25 °C in Bragg–Brentano reflection geometry using a Bruker D2 Phaser XRPD system with Cu Kα radiation (λ = 1.5418 Å) equipped with a LynxEye detector. A standard reference material (NIST 640c) was measured to verify the positions of the diffraction lines and no significant deviation was found. The data were obtained by step scanning in the angle range 10° ≤ 2θ ≤ 80° with a step increment of 0.006° (2θ) and a dwell of 0.25 s per step. A 1 mm slit was used for the measurements.

For the EXAFS study, approximately 0.2 mg of the synthesized radium–barium carbonate, in the form of a number of crystals clustered together, were placed between a few Kapton tape layers and carefully sealed (sample photo is shown in the Supporting Information, Figure S1).

The EXAFS measurements of radium–barium carbonate were performed using the radium L_3_ absorption edge. The data were collected at the wiggler beam line I811 at MAX-lab (Lund University, Sweden), which operated at 1.5 GeV and a maximum current of 180 mA. The EXAFS station was equipped with a Si[111] double-crystal monochromator for the data collection. Higher order harmonics were reduced by detuning the second monochromator crystal to reflect 70% of maximum intensity at the end of the scans. The measurement was performed in transmission and fluorescence modes simultaneously. Ten continuous scans of 10 min each were averaged. The energy calibration was performed by measuring the position of the Pb L_2_ edge of metallic lead before and after the measurement of the radium–barium carbonate sample; the first inflection point of the Pb L_2_ edge of metallic lead was assigned to 15,200 eV.^29^

### 2.4 Solubility Data Collection

The solubility of RaCO_3_ was studied from under- (one sample) and oversaturation (five samples) as a function of ionic strength using NaCl (0.01, 1.3, 1.9, 2.5, 4, and 5 mol·L^–1^) as a background electrolyte. For the undersaturation study, a small sample (approximately 0.5 mg), which was previously measured via XRPD, was transferred from the XRPD sample holder to the test-tube containing 0.5 mL of 0.01 mol·L^–1^ NaCl. For the oversaturation studies, a previously prepared radium stock solution in the form of 2.77 mol·L^–1^ HCl and with a ^226^Ra concentration of 0.40 ± 0.02 mmol·L^–1^ was added to a solution containing Na_2_CO_3_ and NaCl. For all oversaturation samples, the sample volume was 2 mL, the concentration of ^226^Ra was 0.13 mmol·L^–1^ and the concentration of Na_2_CO_3_ was 0.1 mol·L^–1^. Preliminary experiments showed that radium sorption losses on polypropylene at such high radium concentrations were negligible. According to the literature,^30^ the recommended value for the second dissociation constant (p*K*_a2_) of H_2_CO_3_ at 25 °C and zero ionic strength is 10.239. Complete dissociation of H_2_CO_3_ in NaCl media will occur at a lower pH due to activity coefficient changes.^31^ Therefore, the pH was increased by the addition of a small amount of 2 mol·L^–1^ NaOH to both the under- and oversaturation samples.

All polypropylene tubes used for the solubility experiments were pre-washed first with ethanol and then with type 1 Milli-Q water to remove any residues. All samples were gently shaken under a constant temperature of 25.1 ± 0.1 °C using a shaking machine (IKA VXR basic Vibrax) coupled with a heated circulating water bath (Grant Optima T100-P12). After 39 (the undersaturation sample) and 230 (oversaturation samples) days, at least two 100 μL samples were taken and centrifuged in polypropylene tubes at 5 ·10^4^*g* at a constant temperature of 25 °C for 60 min (Beckman Coulter Allegra 64R refrigerated centrifuge with F2402H rotor). The pH of all samples was measured after the last sampling and was always above 12. Radium hydrolysis at this pH is very weak and can be neglected.^16^

After centrifugation, two 10 μL samples were taken and the concentration of ^226^Ra was measured using a High Purity Germanium Detector (Ortec GEM-C5060 coaxial high purity germanium detector 50.5 mm diameter, 68.3 mm length, and 0.9 mm carbon epoxy entrance window coupled to a digital spectrum analyzer Ortec DSPEC50). The detector was calibrated for the same geometry (1 mL of 4 mol·L^–1^ HCl in polypropylene tube) using a mixed radionuclide reference solution (NIST traceable from Eckert and Ziegler, USA). Dead time was always kept below 10%. All gamma spectra obtained were evaluated using the Gamma Vision 7.01.03 software. Radium-226 was measured using its gamma emission peak at 186.2 keV and its half-life, gamma emission energies, and photon emission probabilities were taken from the Decay Data Evaluation Project.^32^

### 2.5 Data Analysis

Refinement of the witherite and radium–barium carbonate XRPD patterns obtained was performed using the Rietveld method^33^ in the Fullprof2k^34^ software package, using both TREOR^35^ and DICVOL-06.^36^ Available witherite unit cell parameters,^37−43^ corrected for the difference in ionic radius between barium and radium,^9^ were used as a starting estimate.

The EXAFS oscillations were extracted from averaged raw data using standard procedures for pre-edge subtraction, spline removal, and data normalization. To obtain quantitative information for the coordination structure of the radium ion, the experimental *k*^3^-weighted EXAFS oscillations were analyzed by non-linear least-squares fits of the data to the EXAFS equation, refining the model parameters: number of backscattering atoms (*N*), mean interatomic distances (*R*), Debye–Waller factor coefficients (σ^2^), and threshold energy (*E*_0_). Data analysis was performed using the EXAFSPAK program package.^44^ Model fitting was performed with theoretical phase and amplitude functions including both single and multiple scattering paths using the *ab initio* FEFF7 code (version 7.02).^45^ Diamond software^46^ was used to visualize the Ra^2+^ surroundings.

The apparent solubilities of RaCO_3_ were derived as follows—first, the free Ra^2+^ concentrations were calculated using values of the measured total Ra^2+^ concentrations, the value of the RaCO_3_ solubility product constant at zero ionic strength obtained from experimental data (undersaturation point at 0.01 mol·L^–1^ was extrapolated to zero ionic strength using the Davies equation), and the value of the estimated stability constant of the RaCO_3_(aq) complex at zero ionic strength taken from the literature (log_10_*K*_0_ = 2.5).^12^ The value of RaCO_3_(aq) stability constant had a very small contribution to the calculated free Ra^2+^ concentration. Then, the free CO_3_^2–^ concentrations were calculated using the value of the total added concentrations of CO_3_^2–^ and the values of the NaCO_3_^–^ stability constants. The values of the NaCO_3_^–^ stability constants were derived via non-linear curve fitting. The derived values were in good agreement with the literature values.^47−59^ Subsequently, the RaCO_3_ solubility product constants were computed as a product of the free Ra^2+^ and free CO_3_^2–^ concentrations and then extrapolated to zero ionic strength using the extended specific ion interaction theory (ESIT). Non-linear curve fitting was performed using a Levenberg–Marquardt iteration algorithm, and the experimental apparent solubility constants of RaCO_3_ were weighted using their standard deviations (ω_*i*_ = 1/σ^2^). Models for activity coefficients computation and adaptation of the ESIT can be found in the Supporting Information.

### 2.6 Uncertainty Assessment

The standard deviations (2σ) of the gamma spectrometric measurements of total Ra^2+^ concentrations were approximately 6%. To the best of our knowledge, the stability constant of RaCO_3_(aq) has never been measured experimentally, and therefore, the standard deviation (2σ) of the stability constant of RaCO_3_(aq) at zero strength was estimated to be 50%. The standard deviation (2σ) of the values of the NaCO_3_^–^ stability constants were estimated based on the fitting results and an extensive literature review.^47−59^ Estimation of uncertainties of stability constants of weak ion pairs including NaCO_3_^–^ is the only reliable method because in this case systematic uncertainties are much greater than stochastic.^16^ The standard deviations (2σ) of the gamma spectrometric measurements, estimated RaCO_3_(aq), and NaCO_3_^–^ stability constants were first propagated to the 2σ standard deviation of free Ra^2+^ and free CO_3_^2–^ concentrations and then to the apparent solubility product constant of RaCO_3_ using standard uncertainty propagation. The value for the RaCO_3_ solubility is subject to some systematic uncertainties due to the probable presence of barium impurities. As a result, the uncertainty (1σ standard deviation of the fit) obtained for the RaCO_3_ solubility at zero ionic strength was increased to reflect possible systematic effects from 0.02 to 0.04 log_10_ units.

The uncertainties reported for the EXAFS refined parameters obtained are 2σ standard deviations related to the least-squares refinements. Variations in the refined parameters obtained using different models and data ranges indicate that the accuracy of the distances given for the separate complexes is within an interval of 0.005–0.02 Å, which is typical for well-defined interactions.

## 3 Results

### 3.1 Crystal Structure of Radium–Barium Carbonate and Its *Ab Initio* Modeling

**Figure 1:**
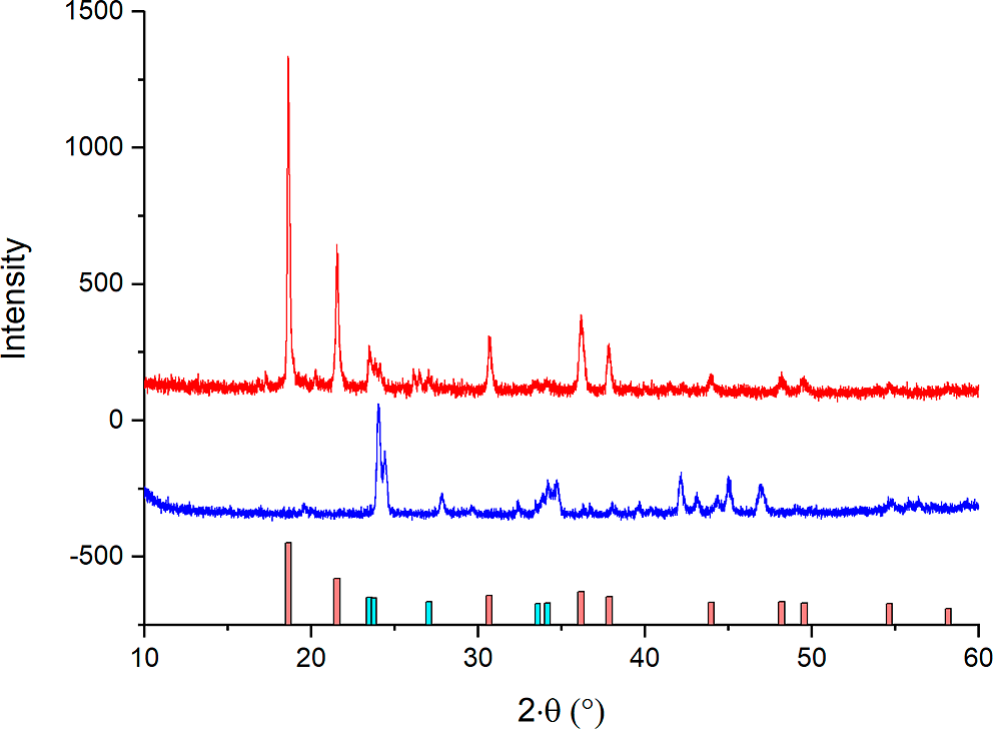
Comparison of the measured XRPD patterns of radium–barium carbonate (upper red line) and witherite (lower blue line) synthesized using the same method.

Rietveld refinement of the XRPD pattern of synthesized BaCO_3_ shows that it is orthorhombic witherite and crystallizes in the *Pnma* (no. 62) space group. The unit cell parameters obtained are in good agreement with the literature values.^37−43^ A comparison of the XRPD patterns of witherite and radium–barium carbonate synthesized using the same method is shown in Figure 1. As can be observed in Figure 1, the diffraction peaks in the radium–barium carbonate XRPD pattern (red) at 23.5 and 23.8° (2θ) have the same shape as the diffraction peaks in the witherite XRPD pattern (blue), but they are slightly shifted to lower angles. Similar observations can be seen for the diffraction peaks in the radium–barium carbonate XRPD pattern at 27.0, 33.6, and 34.2° (Figure 1). The intensity of these diffraction peaks is much lower than the intensities of the other diffraction peaks at 18.6, 21.5, 30.7 36.2, and 37.9° (Figure 1). Thus, it can be concluded that the synthesized radium–barium carbonate contains two phases—a dominating major phase represented by ten diffraction peaks and a minor phase represented by five diffraction peaks. The small systematic shift of the five diffraction peaks in the radium–barium XRPD pattern at 23.5, 23.8, 27.0, 33.6, and 34.2° in comparison to the diffraction peaks of pure witherite shows that the minor phase is isostructural with witherite. The small systematic shift of these peaks to lower angles also shows that the minor orthorhombic Ba(Ra)CO_3_ phase has slightly larger unit cell dimensions than witherite. This is consistent with the fact that the effective ionic radius of radium is 0.06–0.09 Å larger than that of barium, depending on the coordination number.^9^ Similar differences are observed for barite and RaSO_4_.^28^

The limited number of weak diffraction lines makes it impossible to refine any unit cell dimensions for the orthorhombic witherite-type minor phase but it can be concluded that the minor orthorhombic Ba(Ra)CO_3_ phase is isostructural with witherite and crystallizes in the space group *Pnma* (no. 62) with slightly larger unit cell dimensions. Furthermore, it is not possible to determine precisely the barium and radium content in this minor orthorhombic phase, but integration and comparison of the two most intense witherite peaks (blue) at 24.1 and 24.4° (2θ) in Figure 1 with those from the radium–barium carbonate XRPD pattern (red) at 23.5 and 23.8° shows that the peak areas at 23.5, 23.8 and 24.1, 24.4° are equal to 36, 22 and 116, 59, respectively. Taking into account that sample sizes of radium–barium carbonate and pure witherite were similar and that the initial Ra/Ba molar ratio was 0.76:0.24, it can be concluded that almost all barium present in the radium–barium carbonate sample precipitated in the orthorhombic witherite phase. Moreover, the significant displacement (0.6°) of the 23.5 and 23.8° peaks and their relatively high intensity indicates that the minor witherite phase contains a significant amount of Ra. Based on the fact that the intensities of the 23.5 and 23.8° peaks from the radium–barium XRPD pattern are approximately equal to one-third of the intensities of the 24.1 and 24.4° peaks from the pure witherite XRPD pattern, it can be assumed that the approximate stoichiometry of the minor phase is Ba_0.7_Ra_0.3_CO_3_ and that the major phase is almost pure radium carbonate.

The major Ra(Ba)CO_3_ phase is represented by the high-intensity diffraction peaks at 18.6, 21.5, 30.7, 36.2, and 37.9° (Figure 1). The only two sparingly soluble metal ion carbonates in the system used in the synthesis are witherite and RaCO_3_. The radium concentration in the initial system was significantly higher than that of barium (the Ra/Ba molar ratio was 0.76:0.24 for the mixture) and taking into account that almost all Ba precipitated in the minor orthorhombic witherite phase, it can be concluded that the major Ra(Ba)CO_3_ phase is almost pure RaCO_3_ with a Ba/Ra molar ratio of 0.1 or less.

The limited number of reflections did not allow for a direct Rietveld refinement of the major Ra(Ba)CO_3_ phase structure. Attempts to solve the major Ra(Ba)CO_3_ phase by direct space methods were made using the FOX software.^60^ The model consisted of one radium atom and a carbonate ion modeled with the Avogadro software^61^ and fed into FOX as a rigid molecular unit. These two objects were randomly placed and adjusted in a Monte Carlo procedure to obtain an optimal fit between the observed data and the data calculated from the present model. A dynamic occupancy correction was used for modeling the close contact and overlap of different atoms. The resulting final model showed enormous disorder features. Structural disorder in the major Ra(Ba)CO_3_ phase results in a higher symmetry of the unit. A low symmetry cubic space group, *F*23 (no. 196), was used to not impose any additional restraints than cubic symmetry. It must be emphasized that it is hardly possible to estimate the space group with complete certainty for a sample with as few peaks as the phase investigated because of the small sample size and also the small unit cell size. However, only four formula units are required to fill the unit cell; thus, it can be modeled acceptably using the cubic *F*23 space group. Further details of the attempts to solve the crystal structure of Ra(Ba)CO_3_ are given in the Supporting Information.

### 3.2 Interatomic Distances in Radium–Barium Carbonate

**Figure 2:**
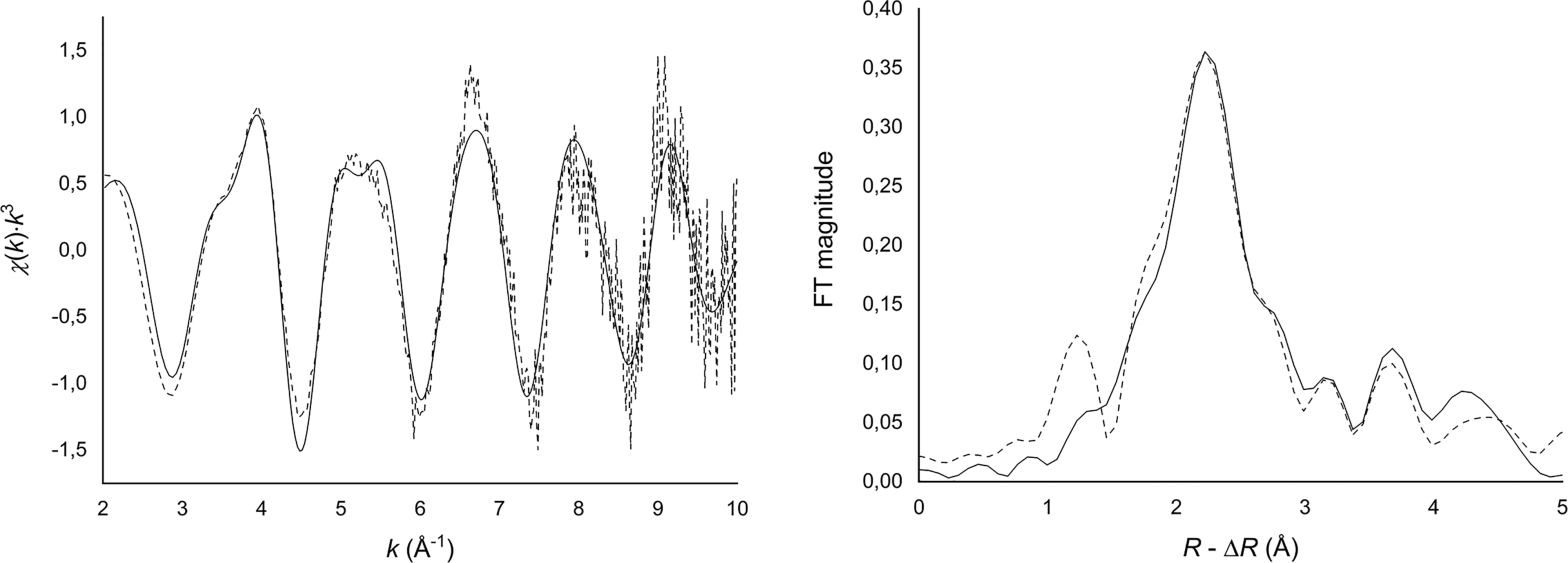
Fit (left) and Fourier transform (right) of raw EXAFS data of the major Ra(Ba)CO_3_ phase. Dashed line—experimental and solid line—calculated.

The quality of the EXAFS data for the mixture of the major Ra(Ba)CO_3_ and minor Ba(Ra)CO_3_ is very good despite the small amount of non-homogenized sample. Based on the XRPD pattern, it can be inferred that the derived Ra–O bond distance in the sample is dominated by the major Ra(Ba)CO_3_ phase and that the influence of the minor Ba(Ra)CO_3_ phase is limited. The results of the EXAFS data refinement are listed in Table 2 and the fits are shown in Figure 2.

**Table 2:** EXAFS Refinement Parameters for the Ra L_3_ EXAFS of the Major Ra(Ba)CO_3_ Phase^a^

	number of backscattering atoms	mean interatomic distance for single scattering paths, *R* (Å)	Debye–Waller factor, σ^2^ (Å^2^)	threshold energy, *E*_0_ (eV)	amplitude reduction factor, (*S*_0_^2^)
Ra–O	9	2.885(5)	0.0144(9)	15,452.8(3)	0.80(6)
Ra–C	6	3.283(3)	0.0090(6)		
Ra–O_II_	6	4.26(1)	0.014(1)		
Ra–O_III_	12	4.94(4)	0.016(2)		

a All uncertainties are 1σ standard deviations related to the least-squares refinements.

The EXAFS data reveal a mean Ra–O bond distance of 2.885(5) Å (1σ), which indicates that radium is surrounded by nine oxygen atoms from the carbonate ions in a broad bond distance distribution in solid Ra(Ba)CO_3_. This is in good agreement with the corresponding mean metal–oxygen distances in strontianite, witherite, and cerussite, as shown in Table 3. The effective ionic radius of Ra^2+^ in a 9-fold coordination can be calculated using the mean Ra–O distance from this work and an atomic radius of the carbonate oxygen of 1.34 Å from Beattie and co-workers^62^ with an estimated 1σ of 0.005 Å and compared with the effective ionic radii of Sr^2+^, Pb^2+^, and Ba^2+^ (Table 3).

**Table 3:** Comparison of Experimental, Mean Metal–Oxygen Distances in Cerussite, Strontianite, Witherite, Ra(Ba)CO_3_, and RaSO_4_ and Effective Ionic Radii of Sr^2+^, Pb^2+^, Ba^2+^, and Ra^2+^ in 9-fold and 12-Fold Coordination

compound	formula	mean metal–oxygen distance^a^ (Å)	metal coordination number	effective ionic radius^b^ (Å)	effective ionic radius^c^ (Å)^9^	references
cerussite	PbCO_3_	2.696	9	1.356	1.35	(38,39, 43, 63,–66)
strontianite	SrCO_3_	2.645	9	1.305	1.31	(38,41,43,67,–69)
witherite	BaCO_3_	2.807	9	1.467	1.47	(37–43)
radium carbonate	Ra(Ba)CO_3_	2.885(3)	9	1.545(6)		EXAFS study, this work
radium nitrate solution	Ra(NO_3_)_2_	2.87(6)	9.2(1.9)	1.53(6)		EXAFS study^11^
radium–barium sulfate	Ra_0.76_Ba_0.24_SO_4_	2.96(2)	12	1.62(2)	1.7	EXAFS study^10^
radium sulfate	RaSO_4_	3.02	12	1.68	1.7	XRPD and DFT study^28^

a Calculated mean metal–oxygen bond distance from the crystal structures in the references.

b Ionic radius of the metal ion calculated by subtracting 1.34 Å^62^ from the mean metal–oxygen bond distance in the reported compounds.

c Ionic radii proposed by Shannon.^9^ Uncertainties are 1σ standard deviations.

As shown in Table 3, the mean metal–oxygen distances in strontianite, witherite, cerussite, and Ra(Ba)CO_3_ are in excellent agreement with the ionic radii for the corresponding metal ions in 9-fold coordination proposed by Shannon,^9^ as well as with the atomic radius of the carbonate oxygen (1.34 Å)proposed by Beattie and co-workers.^62^ Moreover, the ionic radius of Ra^2+^ with the coordination number of 9(2) measured in 0.001 mol·L^–1^ of HNO_3_ via EXAFS^11^ is also in good agreement with the ionic radius of Ra^2+^ in 9-fold coordination measured in this work (1.53 and 1.545 Å, respectively).

The other experimental EXAFS study of a radium compound was conducted by Hedström and co-workers,^10^ who studied a RaSO_4_ sample via XRPD and EXAFS. Hedström et al.^10^ assumed that the studied RaSO_4_ sample was pure but later Matyskin et al.^28^ showed that there was a barium impurity, and the actual stoichiometry of the studied sample was Ra_0.76_Ba_0.24_SO_4_. EXAFS measurements give distances only between the absorbing atom (Ra) and the surrounding atoms (O), therefore the presence of the barium impurity did not affect the derived mean Ra–O bond distance.

As shown in Table 3, the mean Ra–O distance and effective ionic radius of Ra^2+^ in 12-fold coordination, obtained by Hedström et al.,^10^ are slightly larger, as expected, and are in good agreement with the Ra–O distance and with the effective ionic radius of Ra^2+^ in 9-fold coordination obtained in this work. Later, in 2017, Matyskin et al.^28^ studied the crystal structure of a radium sulfate sample of the same origin as Hedström et al.^10^ also via XRPD and the derived unit cell parameters from both studies were the same. However, Matyskin et al.^28^ showed that the actual stoichiometry of the studied sample was Ra_0.76_Ba_0.24_SO_4_; therefore, the obtained unit cell parameters were extrapolated to the unit cell parameters of pure RaSO_4_ using Vegard’s law, and density functional theory (DFT) was used to derive the atomic coordinates and Ra–O distances in pure RaSO_4_.^28^ The derived mean Ra–O bond distance and effective ionic radius of Ra^2+^ in 12-fold coordination are also in good agreement with the data obtained in this work and with the effective ionic radius estimated by Shannon^9^ but is slightly larger than the data obtained by Hedström et al.^10^ (Table 3). The most likely reason for the slightly shorter Ra–O bond distance derived from the EXAFS study is a possible asymmetric bond distance distribution causing the peak in the Fourier transform to be at a slightly shorter distance than the half-height center of the peak. However, the quality of the EXAFS data was not sufficient to perform detailed analysis. Moreover, the quality of the EXAFS data obtained by Hedström et al.^10^ permitted only derivation of the mean Ra–O distance in RaSO_4_, while the EXAFS data obtained in this work were of a significantly higher quality, allowing accurate extraction of longer Ra–O, Ra–C, Ra–O_II_, and Ra–O_III_ distances (Table 2).

The effective ionic radius of Ra^2+^ in 9-fold coordination derived in this work (1.545(6) Å) is also in very good agreement with the radius of Ra^2+^ in 8-fold coordination theoretically estimated by Shannon^9^ (1.48 Å). Moreover, the measured mean Ra–O distance (2.885(3) Å with coordination number 9) can be compared with the Ra–O distance in the first hydration shell obtained via molecular dynamics simulations (2.93 Å with coordination number 9.8^70^ and 2.85 Å with coordination number 8.1^71^).

**Figure 3:**
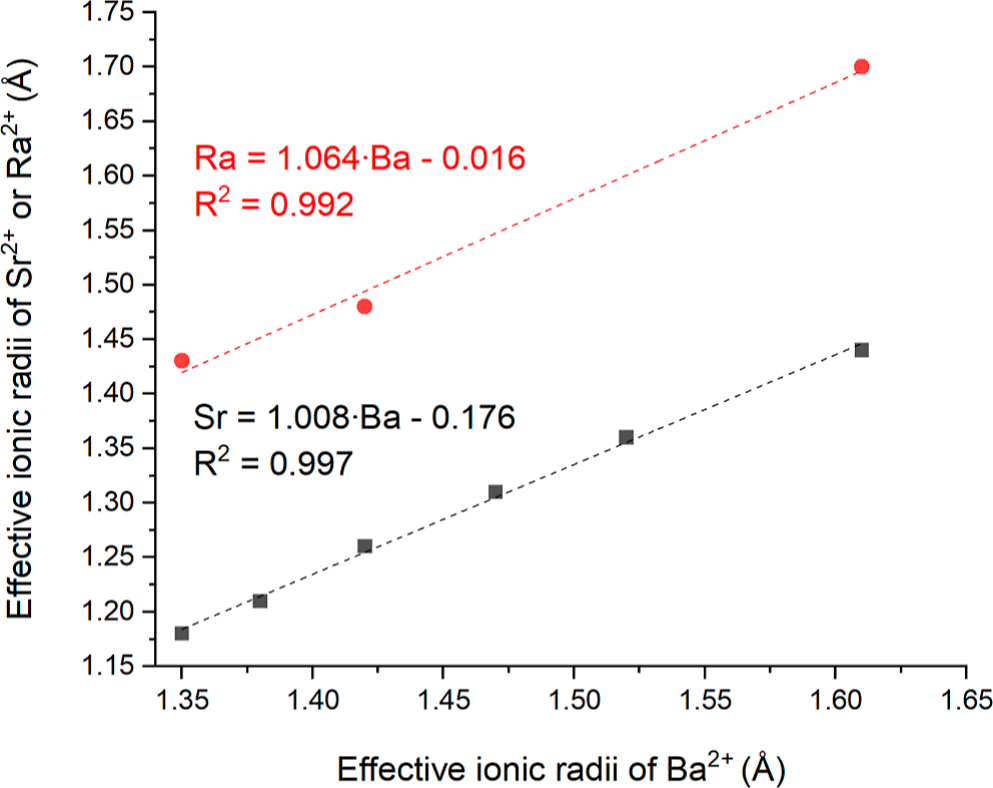
Correlation of effective ionic radii of Ba^2+^ with ionic radii of Sr^2+^ and Ra^2+^ using the literature data.^9,72,73^

The effective ionic radius of Ra^2+^ in 9-fold coordination can be also estimated using the effective ionic radii of Ba^2+^ (Figure 3). As shown in the figure, the ionic radius of Sr^2+^ at each coordination number correlates very well with the corresponding ionic radius of Ba^2+^ at the same coordination number (black, lower line) and this linear correlation is observed over a large range of coordination numbers (from 6 to 12). The same methodology can be applied to derive the effective ionic radius of Ra^2+^ in 9-fold coordination using the available literature data for the ionic radii of Ba^2+^ and Ra^2+^ from Shannon^9^ and the ionic radius of Ra^2+^ in 6-fold coordination from Ahrens,^72^ as listed by Shannon and Prewitt.^73^ The correlation of Ba^2+^ and Ra^2+^ ionic radii result in an effective ionic radius of Ra^2+^ in 9-fold coordination of 1.547 Å, which is within the 1σ standard deviation of the value measured in this work (1.545(6) Å as listed in Table 3).

### 3.3 Solubility of Radium–Barium Carbonate

In determining the solubility, thirty-one solutions were sampled and on every occasion a few samples (3–6) were taken from the solution at each ionic strength. The activity of ^226^Ra at each ionic strength was always within 10% with only one outlier. Most of the samples were centrifuged at 5·10^4^*g*, and the activity of ^226^Ra was similar (within 10%) for both centrifuged and non-centrifuged samples. This indicates that Ra(Ba)CO_3_ does not form colloids. The measured and computed concentrations of total and free Ra^2+^, respectively, are listed in Table 4 (more experimental details and details about extrapolation to zero ionic strength are given in the Supporting Information).

**Table 4:** Measured Total and Computed Free Ra^2+^ Concentrations^a^

ionic strength (mol·kg^–1^)	measured concentration of total Ra^2+^ (mol·L^–1^)	concentration of free Ra^2+^ (mol·L^–1^)
0.01 ± 0.001	(2.65 ± 0.13) × 10^–4^	(2.65 ± 0.13) × 10^–4^
1.34 ± 0.01	(1.19 ± 0.06) × 10^–4^	(1.09 ± 0.08) × 10^–4^
1.98 ± 0.01	(1.23 ± 0.06) × 10^–4^	(1.13 ± 0.08) × 10^–4^
2.65 ± 0.01	(1.14 ± 0.06) × 10^–4^	(1.05 ± 0.08) × 10^–4^
4.38 ± 0.01	(1.22 ± 0.06) × 10^–4^	(1.12 ± 0.08) × 10^–4^
5.59 ± 0.01	(1.19 ± 0.12) × 10^–4^	(1.10 ± 0.13) × 10^–4^

a All uncertainties are 2σ standard deviations. The pH of all samples was measured after the last sampling and was always above 12. The ionic strength of NaCl was recalculated from molar to molal units using the densities and relevant conversion factors.^75^

A comparison of the total and free Ra^2+^ concentrations listed in Table 4 shows that the difference between the two values is less than 1·10^–5^ mol·L^–1^, which means that the formation of the RaCO_3_(aq) complex does not significantly decrease the concentration of total Ra^2+^. As also shown in Table 4, the concentrations of Ra^2+^ (total and free) are within 2σ standard deviations for all ionic strengths above and equal to 1.34 mol·kg^–1^. Similar behavior is observed in the case of the solubility of aragonite (CaCO_3_), strontianite, and witherite in NaCl media.^74^

There is ample evidence in the literature that a weak NaCO_3_^–^ ion pair is formed in aqueous media.^47−59^ According to Marcus and Hefter,^76^ dielectric relaxation spectroscopy has unusual capabilities for studying ion pairing phenomena. The method is particularly sensitive to very weakly associated ion pairs (log_10_*K*° < 1) and can be used to distinguish between various types of ion pairs (solvent separated, solvent shared, and contact ion pairs). Dielectric relaxation spectroscopy was used by Capewell and co-workers^48^ to determine weak NaCO_3_^–^ ion pairing in aqueous CsCl media. It was shown that the apparent stability constant of the NaCO_3_^–^ ion pair in aqueous chloride media is equal to approximately 0.3 at 1 mol·L^–1^ (i.e., *K*_A_ ≈ 0.3) and then decreases with increasing ionic strength. The same trends and similar values for the NaCO_3_^–^ stability constants were obtained in potentiometric^49,58^ and spectroscopic^56−59^ studies. However, weak ion pairing is always subject to some uncertainties, mostly systematic, due to various effects: ion pair formation between components of the background medium, separation of short-range ion interaction, and weak ion pairing, among others.^16^ Therefore, relatively high uncertainties were assigned to all values of the NaCO_3_^–^ stability constant obtained by numerical fitting, despite good agreement between the values obtained in this work and the literature values. The values of the apparent NaCO_3_^–^ stability constant and associated uncertainties obtained in this work and reported by Capewell and co-workers,^48^ who studied NaCO_3_^–^ ion pairing by dielectric relaxation spectroscopy (also in chloride media), are listed in Table 5.

**Table 5:** Stability Constants of the NaCO_3_^–^ Ion Pair from This Work and the Literature and Computed Free CO_3_^2–^ Concentration^a^

ionic strength (mol·kg^–1^)	stability constant of NaCO_3_^–^ ion pair	stability constant of NaCO_3_^–^ ion pair from Capewell et al.^48^	concentration of free CO_3_^2–^ (mol·L^–1^)
1.34 ± 0.01	0.4 ± 0.2	0.3	0.066 ± 0.035
1.98 ± 0.01	0.1 ± 0.05	0.1	0.081 ± 0.034
2.65 ± 0.01	0.04 ± 0.02	0.05	0.091 ± 0.047
4.38 ± 0.01	0.002 ± 0.001	<0.01	0.010 ± 0.044
5.59 ± 0.01	0.0003 ± 0.0002	<0.01	0.010 ± 0.062

a All uncertainties are 2σ standard deviations. The pH values of all samples were measured after the last sampling and were always above 12. The ionic strength of NaCl was recalculated from molar to molal units using the densities and relevant conversion factors.^75^

**Figure 4:**
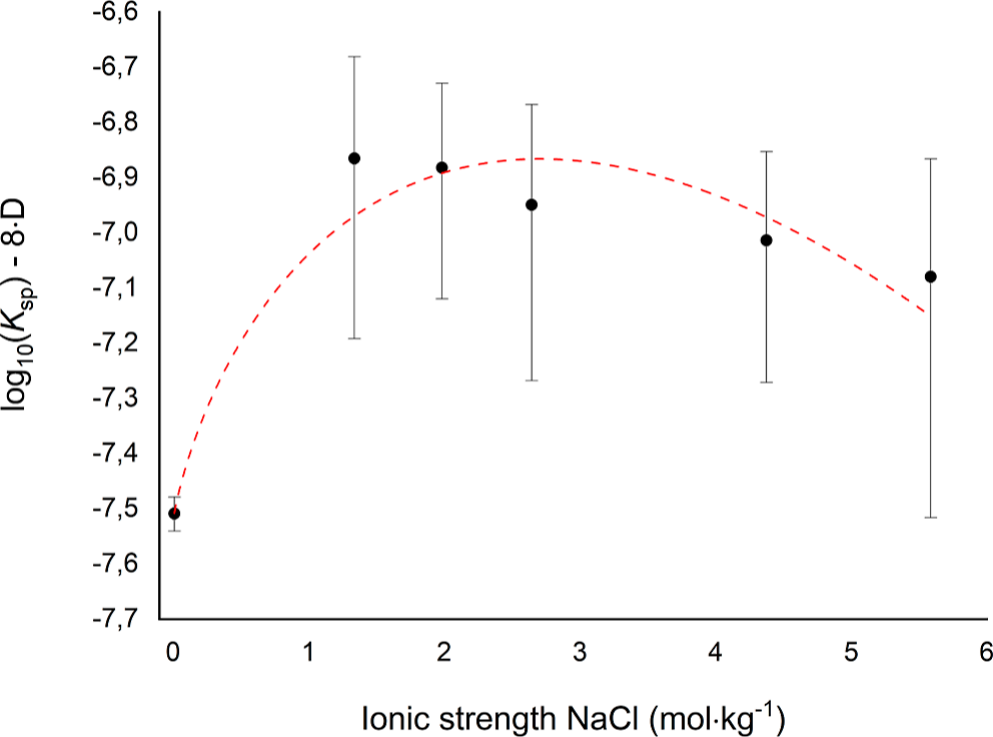
Extrapolation of experimental log_10_*K*_sp_ of Ra(Ba)CO_3_ to zero ionic strength using the ESIT. Error bars are 2σ standard deviations.

As shown in Table 5, NaCO_3_^–^ ion pairing will only affect the concentration of free CO_3_^2–^ at the first three ionic strengths and the NaCO_3_^–^ stability constants at ionic strengths above 4 mol·kg^–1^ are too small to decrease the concentration of free CO_3_^2–^ in its complex formation with Na^+^. The apparent solubility product constants of Ra(Ba)CO_3_ were calculated as the product of the free Ra^2+^ and free CO_3_^2–^ concentrations (Tables 4 and 5, respectively) and extrapolation of the Ra(Ba)CO_3_ apparent solubility product constant to zero ionic strength using the ESIT is shown in Figure 4. All parameters obtained in the regression analysis are listed in Table 6.

**Table 6:** Solubility Product of Ra(Ba)CO_3_ and Ra^2+^–Cl^–^ Ion Interaction Coefficients (ε) at 25 °C and Zero Ionic Strength^a^

constant	value	references
log_10_ *K*_sp_^0^ of Ra(Ba)CO_3_	–7.52 ± 0.02	this work—Davies equation
log_10_ *K*_sp_^0^	–7.52 ± 0.02	this work—ESIT
ε_1_ (Ra^2+^, Cl^–^)	–0.49 ± 0.05	
ε_2_ (Ra^2+^, Cl^–^)	0.56 ± 0.07	

a All uncertainties are 1σ standard deviations.

As shown in Table 6, extrapolation of the Ra(Ba)CO_3_ apparent solubility product constant using the Davies equation and ESIT leads to the same value for the Ra(Ba)CO_3_ solubility product at zero ionic strength.

**Figure 5:**
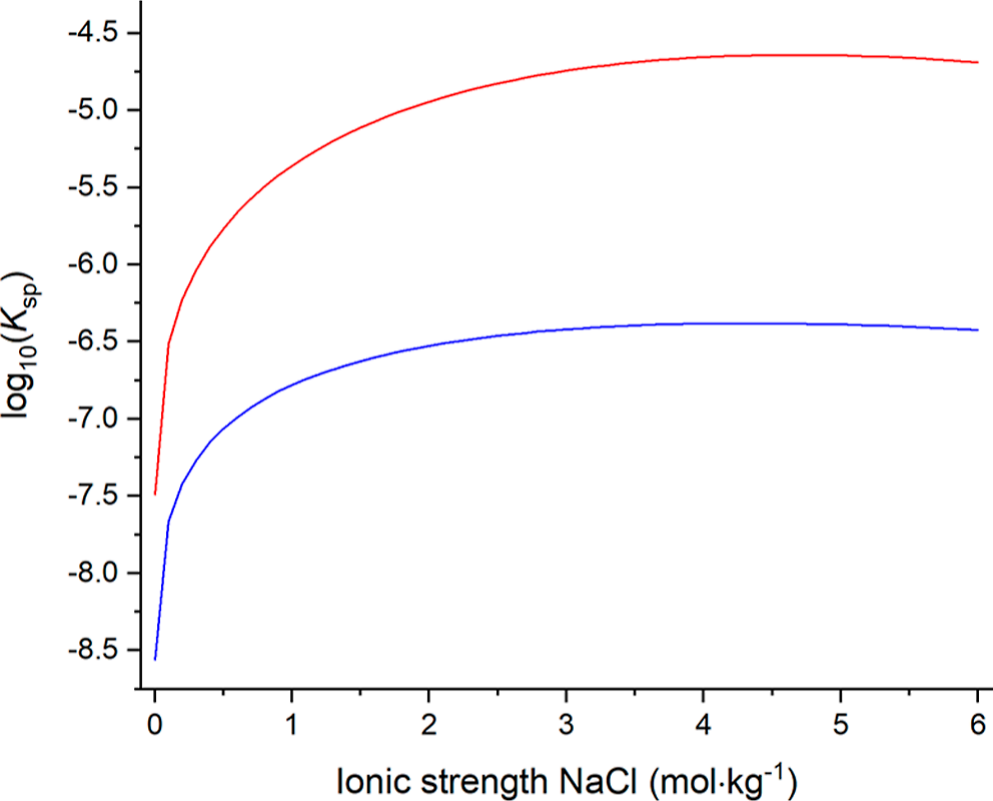
Comparison of the logarithm of the apparent RaCO_3_ (this work) and BaCO_3_ (witherite, from Millero et al.^74^) solubility products at different ionic strengths of aqueous NaCl media at 25 °C.

Millero and co-workers^74^ studied the solubility of witherite in NaCl media as a function of ionic strength, and these experimental data were used to derive ε_1_ (Ba^2+^, Cl^–^) and ε_2_ (Ba^2+^, Cl^–^) ESIT ion interaction coefficients. The derived ESIT coefficients were used to plot the witherite solubility product as a function of NaCl ionic strength and the increase of its solubility product with an increase of the NaCl concentration is compared with that of Ra(Ba)CO_3_ in Figure 5.

As shown in Figure 5, the shape of the solubility product curves of Ra(Ba)CO_3_ and witherite in aqueous NaCl media are very similar, which means that Ra^2+^ and Ba^2+^ have similar activity coefficients and undergo similar short-range ion interactions in aqueous NaCl media. The same conclusion was obtained by Matyskin and co-workers who studied the hydrolysis of Ra^2+^ and Ba^2+^ in aqueous NaClO_4_–NaOH media,^16^ the complex formation of these metal ions with ethylenediaminetetraacetic acid (EDTA) also in aqueous NaCl media,^17^ and the solubility product of RaSO_4_ in aqueous NaCl media.^77^

## 4 Discussion

The results obtained in the crystallographic study of this work are different to the results obtained by Weigel and Trinkl^13^ and Butkalyuk and co-workers,^14^ who studied RaCO_3_ by XRPD and reported that it is isostructural with witherite. Weigel and Trinkl^13^ precipitated RaCO_3_ by addition of (NH_4_)_2_CO_3_ (p.a. grade, Merck) to approximately 65 μg of RaCl_2_ dissolved in aqueous solution (obtained from RaBr_2_ with 98–99% purity), while Butkalyuk and co-workers^14^ synthesized RaCO_3_ by long heating of Ra(NO_3_)_2_ (with a metallic purity of 99% analyzed by inductively coupled plasma-optical emission spectroscopy) in a Ni crucible (99.99% purity). In both papers, the RaCO_3_ sample was prepared by calcination (3.5 h at 640 °C, and 8 h at 800 °C, respectively), and the purity of the synthesized samples was not measured. In the case of BaCO_3_ and SrCO_3_, a phase transformation from the space group *Pnma* (orthorhombic, no. 62) to *R*3*m* (trigonal, no. 160) occurs at approximately 811^37^ and 912 °C,^78^ respectively, and this difference may be explained by a possible phase transformation. A similar transformation may occur for RaCO_3_ at temperatures above 250 °C.

Another possible explanation is that the Ra(Ba)CO_3_ co-precipitate crystallizes in a space group different from the space group of pure RaCO_3_ or witherite due to Ba^2+^ or SO_4_^2–^ doping. In many cases, doping can result in the formation of a compound with a crystal structure different from the crystal structure of the pure end members and can also decrease the phase transformation temperatures. An example of SO_4_^2–^ doping was described by Nishino and co-workers.^79^ They reported that mixing witherite doped with up to 10 mol % of barite (BaSO_4_) and heating to 820 °C for 30 min results in the formation of monoclinic BaCO_3_ that is stable at room temperature and atmospheric pressure. However, in this work, witherite was synthesized using the same method as Ra(Ba)CO_3_ and a typical orthorhombic witherite crystal structure was obtained. This indicates that the possible presence of small amounts of SO_4_^2–^ had a negligible influence on the crystal structure of the obtained Ra(Ba)CO_3_ phase. Moreover, it can be shown that the synthesis of non-orthorhombic BaCO_3_,^37,38,40,42^ SrCO_3_,^42^ and PbCO_3_^65,80^ requires high pressures and temperatures, far above 250 °C.

An example of witherite doping with Ca^2+^ and Sr^2+^ has been reported in a study by Lander,^78^ who synthesized barium (46 wt %), strontium (46 wt %), and calcium (8 wt %) carbonate by co-precipitation from a solution and measured the triple carbonate obtained using XRPD. Lander found that the synthesized co-precipitate was orthorhombic with a crystal structure similar to aragonite. A systematic study of the crystal structures of Ba_1–*x*_Sr_*x*_CO_3_ co-precipitates was performed by Weinbruch and co-workers.^81^ They synthesized Ba_1–*x*_Sr_*x*_CO_3_ co-precipitates of different compositions (14 samples in total) by grinding and mixing pure witherite and strontianite and reported that all co-precipitates had orthorhombic crystal structures at room temperature. This implies that co-precipitation of Ca^2+^, Sr^2+^, and Ba^2+^ results in the formation of stable orthorhombic phases that are isostructural with the pure end-members aragonite, strontianite, and witherite.

In summary, there is evidence in the literature that temperatures far above 250 °C are required to synthesize pure or doped BaCO_3_ with a crystal structure different to witherite.^37−43^ Furthermore, both methods of Ra(Ba)CO_3_ synthesis used in the present work (three cycles of Ra_0.76_Ba_0.24_SO_4_ heating in 1.5 mol·L^–1^ Na_2_CO_3_ to 85 °C, cooling, and subsequent removal of the supernatant and precipitation of Ra(Ba)CO_3_ by the addition of RaCl_2_ solution to highly alkaline Na_2_CO_3_ solution) results in the same values of solubility product, which is approximately 10 times higher than the solubility product of witherite. This shows that the synthesis route of Ra(Ba)CO_3_ has no influence on its solubility product or crystal structure.

The difference in the crystal structure between witherite and the major Ra(Ba)CO_3_ phase obtained in this work can be explained by the fact that both pure RaCO_3_ and the major Ra(Ba)CO_3_ phase crystallize in the same space group (presumably cubic *F*-centered) with exceptional disorder. In this case, the possible barium impurity in the major Ra(Ba)CO_3_ phase obtained would co-precipitate as a minor component and would adopt the crystal structure of pure RaCO_3_. This hypothesis is supported by the significantly higher solubility of the major Ra(Ba)CO_3_ phase determined in this work compared with the solubility of witherite. The decimal logarithm of the witherite solubility product at infinite dilution (log_10_*K*_sp_^0^) is equal to −8.56,^18^ and extrapolation of this value to the solubility product of RaCO_3_ using an electrostatic model and assuming that it is isostructural with witherite (*Pnma* no. 62) gives a value of −8.3.^12^ The larger solubility product of Ra(Ba)CO_3_ obtained in this work (log_10_*K*_sp_^0^ = −7.5) compared to the solubility product of witherite shows that the major Ra(Ba)CO_3_ phase dominates and indicates that this phase is disordered because such phases usually have higher solubilities than the equivalent crystalline phases. The fact that the solubility product of Ra(Ba)CO_3_ in NaCl media obtained from undersaturation (1 sample) and oversaturation (5 samples) is almost one order of magnitude higher than the solubility product of witherite shows that the same disordered Ra(Ba)CO_3_ phase is systematically formed at all ionic strengths. Moreover, the solubility studies of Ra(Ba)CO_3_ performed in this work are in a very good agreement with the findings of Nikitin,^15^ who experimentally studied and compared solubilities of pure RaCO_3_ and BaCO_3_. Nikitin’s experiments were very similar to experiments preformed in this work—he precipitated RaCO_3_ inNH_4_Cl media by the addition of (NH_4_)_2_CO_3_ and NaOH to RaCl_2_ solution (total *I* ≈ 2.7 mol·L^–1^) and then measured the concentration of radium in the aqueous phase. The same experiments were carried out with BaCO_3_, and it was found that the solubility of RaCO_3_ in grams per liter was approximately 10 times higher than the solubility of BaCO_3_ at the experimental conditions used (total *I* ≈ 2.7 mol·L^–1^). Thermodynamic modeling performed by Brown and co-workers,^18^ who assumed that the solubility of each alkaline-earth metal carbonate is a function of the inverse of absolute temperature with a constant, but non-zero, heat capacity change, determined a solubility product for pure RaCO_3_ of log_10_*K*_sp_^0^ = −7.57 (*s* = 0.047 g·L^–1^). Thus, the value of the Ra(Ba)CO_3_ solubility product determined in this work is in excellent agreement with experimental results for pure RaCO_3_ solubility from Nikitin^15^ and the thermodynamic modeling by Brown et al.^18^ and the crystallographic and solubility studies of RaCO_3_ complement each other. Additionally, according to the literature,^82^ the solubility of Ra(NO_3_)_2_ is higher than the solubility of Ba(NO_3_)_2_ which is not within the trend of the solubilities of alkaline-earth metal nitrates. This indicates that a similar phenomenon may occur in Ra(NO_3_)_2_ synthesized at room temperature as was found for RaCO_3_, and both phases have disordered oxygen atoms around the nitrogen and carbon, respectively. Another argument that RaCO_3_ is not isostructural with witherite is the limited co-precipitation of trace Ra^2+^ within witherite. To the best of our knowledge, all reported partition (crystallization) coefficients of radium in witherite are below unity,^26,27^ which means that most of the radium does not co-precipitate with witherite but stays in the aqueous phase.

In summary, the following evidence confirms that the major Ra(Ba)CO_3_ phase obtained in this work behaves as pure RaCO_3_ and presumably crystallizes in an *F*-centered cubic space group with exceptional disorder of the carbonate ion oxygen atoms:

1. High temperatures and pressures are required to obtain pure or doped non-orthorhombic BaCO_3_, and BaCO_3_ synthesized using the same method as Ra(Ba)CO_3_ always crystallizes in the orthorhombic space group.
2. The solubility product of Ra(Ba)CO_3_ measured from both under- and oversaturation (6 measurements) shows that it is 10 times higher than witherite solubility product at zero ionic strength, and this result is consistent with literature data^15^ where the solubility of pure RaCO_3_ was measured and also with thermodynamic modeling.^18^
3. Radium co-precipitation with barium into witherite (orthorhombic BaCO_3_) is very limited, in complete contrast to the almost complete radium co-precipitation with barium into barite (orthorhombic BaSO_4_).
4. The effective ionic radius of Ra^2+^ in 9-fold coordination determined from the EXAFS data (1.545(6) Å) is in excellent agreement with the predicted value (1.547 Å) demonstrating that the major dominant phase is almost pure RaCO_3_.

Table 7 shows the influence of the effective ionic radii of the metal ion on the crystal structure of metal carbonates. The table shows that radium carbonate is the only carbonate which forms disordered crystals at ambient conditions. Possibly, the ionic radius of Ra^2+^ is too large to fit into an ordered orthorhombic crystal system with carbonate ions. Moreover, the ionic radii of Ba^2+^ and Ra^2+^, even though close in magnitude, differ by too much to fit into their respective crystal structures, confirmed by experimental data of limited Ba^2+^ co-precipitation in the major RaCO_3_ phase and limited co-precipitation of Ra^2+^ within witherite.^26,27^ Differences in the crystal structure of RaCO_3_ and witherite and limited Ra^2+^ co-precipitation within witherite suggests that Ra^2+^ is mostly physically absorbed during the crystal growth or at the surface of witherite.

**Table 7:** Influence of Effective Ionic Radii of the Metal Ion on the Crystal Structure of Metal Carbonates at Ambient Conditions

compound	coordination number	effective ionic radii (Å)^9^	crystal system and space group
MgCO_3_	6	0.72	trigonal calcite type, *R*3̅*c* (no. 167)
ZnCO_3_	6	0.74	
CoCO_3_	6	0.745	
FeCO_3_	6	0.78	
MnCO_3_	6	0.83	
CdCO_3_	6	0.95	
CaCO_3_ (calcite)	6	1.0	
CaCO_3_ (aragonite)	9	1.18	orthorhombic aragonite type, *Pnma* (no. 62)
SrCO_3_	9	1.31	
PbCO_3_	9	1.35	
BaCO_3_	9	1.47	
RaCO_3_	9	1.545(6)^a^	disordered, presumably *F*-centered cubic *F*23 (no. 196)

a Effective ionic radius of Ra was measured in this work, and its uncertainty is 1σ standard deviation.

## 5 Conclusions

In this work, a mixture of major Ra(Ba)CO_3_ and minor orthorhombic Ba(Ra)CO_3_ phases, dominated by the former, was synthesized at atmospheric pressure and low temperatures (below 250 °C) and measured by XRPD and EXAFS techniques. It was found that the minor orthorhombic Ba(Ra)CO_3_ phase is isostructural with witherite and crystallizes in the space group *Pnma* (no. 62), with slightly larger unit cell dimensions due to the larger ionic radius of Ra^2+^. Presumably, the major Ra(Ba)CO_3_ phase crystallizes in the *F*-centered cubic space group with exceptional structural disorder of the carbonate ions. The derived bond distance from the EXAFS data reveals that radium is surrounded by nine oxygens from the carbonate ions in a broad bond distance distribution in solid Ra(Ba)CO_3_ with a mean Ra–O bond distance of 2.885(3) Å. The mean Ra–O bond distance is consistent with the literature and gives an effective ionic radius of Ra^2+^ in 9-fold coordination of 1.545(6) Å (1σ). The apparent solubility of RaCO_3_ was experimentally determined as a function of ionic strength over a wide range of NaCl concentrations. It was shown that the RaCO_3_ solubility product is one order of magnitude higher than the solubility product of witherite at all ionic strengths, which confirms that RaCO_3_ synthesized at room temperature is not isostructural with witherite.

## Supporting Information Available

Methods used to prepare RaCO_3_ sample for the EXAFS measurements including photo of the measured sample, details of RaCO_3_ solubility computations including adaptation of the extended interaction theory, and results of the attempt to solve RaCO_3_ crystal structure using Rietveld refinement combined with direct-space ab initio modeling (PDF)

## Notes

All data are available in the paper. Additional data and measured XRD patterns are available on request to the corresponding author.
