# Supplementary material for: Disordered Crystal Structure and Anomalously High Solubility of Radium Carbonate
Source: Inorg Chem. 2023 Jul 21;62(30):12038–49. doi: 10.1021/acs.inorgchem.3c01513 (PMC10394661; doi:10.1021/acs.inorgchem.3c01513)
Supplement: Supplementary file 1 — ic3c01513_si_001.pdf [file ic3c01513_si_001.pdf]

Supplementary information

Disordered crystal structure and anomalously high solubility of radium carbonate

Artem V. Matyskin,[1,2] Burçak Ebin,[1] Stefan Allard,[1] Natallia Torapava,[3,4] Lars Eriksson,[5] Ingmar Persson,[6] Paul L. Brown[7] and Christian Ekberg[1]*

[1] Nuclear Chemistry and Industrial Materials Recycling group, Energy and Materials Division, Department of Chemistry and Chemical Engineering, Chalmers University of Technology, Kemivägen 4, SE-41296 Gothenburg, Sweden

[2] Present address: Radiation Science and Engineering Center, College of Engineering, Pennsylvania State University, 135 Breazeale Nuclear Reactor, University Park, Pennsylvania, 16802, United States of America

[3] MAX IV Laboratory, Lund University, Fotongatan 2, SE-22594 Lund, Sweden

[4] Present address: Talga AB, Södra Kungsgatan 5 B, SE-972 35 Luleå, Sweden

[5] Arrhenius Laboratory, Department of Materials and Environmental Chemistry, Stockholm University, Svante Arrhenius väg 16 C, SE-11691 Stockholm, Sweden

[6] Department of Molecular Sciences, Swedish University of Agricultural Sciences, P.O. Box 7015, SE-75007 Uppsala, Sweden

[7] Rio Tinto Development and Technology, 1 Research Avenue, 3083 Bundoora, Victoria, Australia

[*] Corresponding author: matyskin.artem@gmail.com

1. Methods

For the EXAFS study, approximately 0.2 mg of the synthesized radium-barium carbonate, in the form of a number of crystals clustered together, were placed between a few Kapton tape layers and carefully sealed (Figure S1).

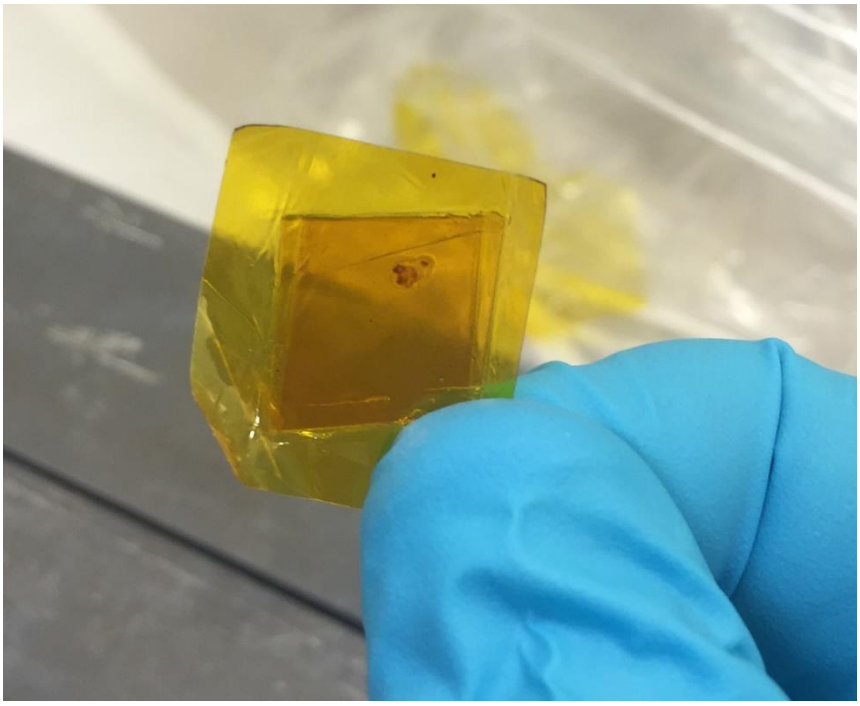

Figure S1. Radium-barium carbonate sample measured via EXAFS

2. Computations

2.1 Models for activity coefficients computation

The Davies equation[1] can be used to calculate activity coefficients of an individual ion *i* at an ionic strength below approximately 0.3 mol·kg^{-1}:

$$\log_{10} \gamma_i = -z_i^2 \cdot A \cdot \frac{\sqrt{I_m}}{1+\sqrt{I_m}} - 0.2 \cdot I_m \tag{1}$$

where z_i is the charge of ion *i*, *A* is a temperature and solvent permittivity dependent constant which is equal to 0.5093 mol$^{-0.5}$·L$^{0.5}$ for aqueous solutions at 25 °C and I_m is the ionic strength in mol·kg^{-1}.

The specific ion interaction theory (SIT) developed by Brønsted[2-3], Scatchard[4] and Guggenheim and Turgeon[5] can be used to calculate activity coefficients of an individual ion *i* at ionic strengths below approximately 3.5 mol·kg^{-1}:

$$\log_{10} \gamma_i = -z_i^2 \cdot D_H + \sum_j \varepsilon(i,j,I_m) \cdot m_j \tag{2}$$

where D_H is the Debye–Hückel term, $\varepsilon(i, j, I_m)$ is the interaction coefficient of ion i with all oppositely charged ions j and m_j is the molal concentration of ion j. The Debye–Hückel term is defined as:

$$D_H = \frac{A \cdot \sqrt{I_m}}{1 + B \cdot a \cdot \sqrt{I_m}} \qquad (3)$$

where B is the temperature and solvent permittivity dependent constant and a is the distance of closest approach, which is the minimal distance at which two ions can approach each other. The distance of closest approach a depends on the nature of the ions i and j, their concentrations, solvent and the nature and concentrations of other species present in the studied system[6]. In this work, the product of $B \cdot a$ was fixed at a value of 1.5.

The extended specific ion interaction theory (ESIT), developed by Ciavatta[7] can be used to calculate activity coefficients of an individual ion i at high ionic strength (up to saturation in some cases):

$$\log_{10} \gamma_i = -z_i^2 \cdot D_H + \sum_j \left(\varepsilon_1(i, j, I_m) + \varepsilon_2(i, j, I_m) \cdot \log_{10} I_m\right) \cdot m_j \qquad (4)$$

where $\varepsilon_1(i, j, I_m)$ and $\varepsilon_2(i, j, I_m)$ are the first and second interaction coefficient of ion i with all oppositely charged ions j respectively.

2.2 Adaptation of the extended ion interaction theory

The solubility reaction for solid $RaCO_3$ is given by the equilibrium reaction:

$$\mathrm{RaCO_3(s)} \rightleftharpoons \mathrm{Ra^{2+}} + \mathrm{CO_3^{2-}} \qquad (5)$$

The equilibrium solubility product constant at infinite dilution for reaction (5) can be calculated using the following equation:

$$K_{SP}^0 = [\mathrm{Ra^{2+}}]^{free} \cdot \gamma_{\mathrm{Ra^{2+}}} \cdot [\mathrm{CO_3^{2-}}]^{free} \cdot \gamma_{\mathrm{CO_3^{2-}}} \qquad (6)$$

The concentration of Na_2CO_3 was 100 $mM \cdot L^{-1}$ for all oversaturation samples and was much lower compared to the total ionic strength of the NaCl electrolyte. Therefore, it can be assumed that Na_2CO_3 had a small contribution to the ionic medium and ion interactions of the carbonate ion with all positively charged ions was minimal[8-10]. There is agreement in the literature that the alkaline-earth metal ions form complexes with carbonate ions in aqueous media[11-12]:

$$\mathrm{Ra^{2+}} + \mathrm{CO_3^{2-}} \rightleftharpoons \mathrm{RaCO_3(aq)} \qquad (7)$$

Therefore, in the case of the oversaturation samples, the *total* measured Ra^{2+} concentration will be equal to the sum of the *free* Ra^{2+} and $RaCO_3$(aq). The equilibrium constant at infinite dilution for the reaction (7) is:

$$K_{IP}^{0} = \frac{[\mathrm{RaCO}_3^{aq}] \cdot \gamma_{\mathrm{RaCO}_3^{aq}}}{[\mathrm{Ra}^{2+}]^{free} \cdot \gamma_{\mathrm{Ra}^{2+}} \cdot [\mathrm{CO}_3^{2-}]^{free} \cdot \gamma_{\mathrm{CO}_3^{2-}}} \quad (8)$$

Combination of Eqs 6 and 8 gives:

$$[\mathrm{RaCO}_3(aq)] = K_{IP}^{0} \cdot K_{SP}^{0} \quad (9)$$

assuming that $\gamma_{\mathrm{RaCO}_3^{aq}}$ is equal to unity.

As is shown in Eq. 9 and in the literature[13], the concentration of $RaCO_3$(aq) does not depend on ionic strength and is equal to the product of the radium carbonate solubility product constant and the stability constant of $RaCO_3$(aq) at zero ionic strength. Recently, it was shown that radium hydrolysis is weak, and can be neglected in approximately 0.1 mol·L^{-1} NaOH[14]. Therefore, the *total* measured radium concentration was corrected for $RaCO_3$(aq) using the following equation:

$$[\mathrm{Ra}^{2+}]^{free} = [\mathrm{Ra}^{2+}]^{total} - K_{IP}^{0} \cdot K_{SP}^{0} \quad (10)$$

There is evidence that the sodium ion forms a weak ion pair with the carbonate ion in aqueous media[15-27]:

$$\mathrm{Na}^{+} + \mathrm{CO}_3^{2-} \rightleftharpoons \mathrm{NaCO}_3^{-} \quad (11)$$

Consequently, in the case of the oversaturation samples, the *total* CO_3^{2-} concentration will be equal to the sum of the *free* CO_3^{2-} and $NaCO_3^-$. The equilibrium constant at infinite dilution for reaction (11) is:

$$K_{IP}^{apparent} = \frac{[\mathrm{NaCO}_3^{-}]}{[\mathrm{Na}^{+}]^{free} \cdot [\mathrm{CO}_3^{2-}]^{free}} \quad (12)$$

As shown in Eq. 12, the concentration of $NaCO_3^-$ can be expressed as a product of its apparent stability constant and *free* sodium and carbonate concentrations. The concentration of the *free* carbonate ion can be calculated as:

$$[\mathrm{CO}_3^{2-}]^{free} = \frac{[\mathrm{CO}_3^{2-}]^{total}}{1 + K_{IP}^{apparent} \cdot [\mathrm{Na}^{+}]^{free}} \quad (13)$$

The concentration of Na^+ (background electrolyte) was much higher (more than 90 % of all ionic strength) than the concentration of Na_2CO_3 in all oversaturation samples. Therefore, it can be assumed that the concentration of the *free* Na^+ is constant and equal to the ionic strength. The apparent stability constant for the $NaCO_3^-$ ion pair can be obtained both from the literature and in a non-linear regression:

$$\log_{10} K_{IP}^{apparent} - \frac{z_i^2 \cdot A \cdot \sqrt{I_m}}{1 + 1.5 \cdot \sqrt{I_m}} = \log_{10} K_{IP}^{0} - \left(\Delta\varepsilon_1 + \Delta\varepsilon_2 \cdot \log_{10} I_m\right) \cdot [Cl^{-}] \quad (14)$$

where $\Delta\varepsilon_1$ (or $\Delta\varepsilon_2$) is equal to:

$$\Delta\varepsilon_1 = \left(-\varepsilon_1(Na^+, NaCO_3^-) + \varepsilon_1(Na^+, Cl^-) + \varepsilon_1(Na^+, CO_3^{2-})\right) \quad (15)$$

The solubility product constant of $RaCO_3$ at zero ionic strength (Eq. 6) and associated ion interaction coefficients can be obtained via non-linear regression:

$$\log_{10} K_{SP}^{apparent} - \frac{z_i^2 \cdot A \cdot \sqrt{I_m}}{1 + 1.5 \cdot \sqrt{I_m}} = \log_{10} K_{SP}^0 - \left(\Delta\varepsilon_1 + \Delta\varepsilon_2 \cdot \log_{10} I_m\right) \cdot [Cl^-] \quad (16)$$

where $K_{sp}^{apparent}$ is the product of the *free* radium (Eq. 10) and *free* carbonate (Eq. 13) concentrations and $\Delta\varepsilon_n$ ($\Delta\varepsilon_1$ or $\Delta\varepsilon_2$) are equal to:

$$\Delta\varepsilon_n = \left(-\varepsilon_n\left(\mathrm{Ra}^{2+}, \mathrm{Cl}^-\right) + \varepsilon_n\left(\mathrm{Na}^+, \mathrm{CO}_3^{2-}\right)\right) \quad (17)$$

with n equal to 1 or 2.

3. Attempt to solve the crystal structure of Ra(Ba)CO_3

The best outcome of the attempts to solve the crystal structure of the major cubic Ra(Ba)CO_3 phase was a model consisting of one radium atom and a carbonate ion modelled with the Avogadro software[28] and fed into FOX software[29] as a rigid molecular unit. These two objects were randomly placed and adjusted in a Monte Carlo procedure to obtain an optimal fit between the observed data and the data calculated from the present model. A dynamic occupancy correction was used for modelling the close contact and overlap of different atoms. The resulting final model showed enormous disorder features. Structural disorder in the major cubic Ra(Ba)CO_3 phase results in a higher symmetry of the unit. A low symmetry cubic space group, *F23* (no. 196), was used to not impose any additional restraints than cubic symmetry. It must be emphasized that it is hardly possible to estimate the space group with complete certainty for a sample with as few peaks as the phase investigated because of the small sample size and also the small unit cell size. However, only four formula units are required to fill the unit cell, thus it can be modelled acceptably using the cubic *F23* space group.

Rietveld refinement of the major cubic Ra(Ba)CO_3 crystal structure modelled in FOX was made using the Fullprof software package[30]. Soft constraints were applied to the carbonate ion to approximately maintain its triangular shape. An overall isotropic displacement parameter was used, and the occupation parameters were allowed to be refined. Rietveld refinement profile and parameters are shown in Fig. S2 and Table S1, respectively.

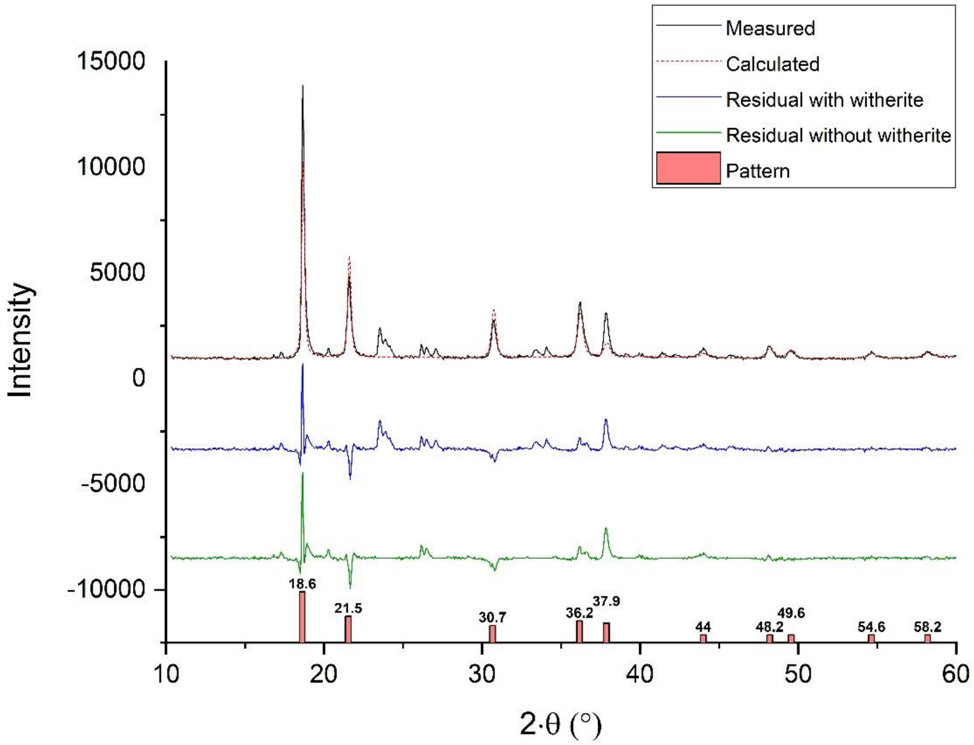

Figure S2. Rietveld refinement profile of the major Ra(Ba)CO_3 phase.

Table S1. Rietveld refinement of the 10 observed reflections of the major Ra(Ba)CO_3 phase.

$h^2+k^2+l^2$	h	k	l	$2\cdot\theta_{obs.}$	$2\cdot\theta_{calc}$	$2\cdot\theta_{diff.}$
3	1	1	1	18.6615	18.6602	0.0013
4	2	0	0	21.5759	21.5791	-0.0032
8	2	2	0	30.7194	30.7035	0.0159
11	3	1	1	36.2005	36.1715	0.0290
12	2	2	2	37.8341	37.8395	-0.0054
16	4	0	0	43.9458	43.9750	-0.0292
19	3	3	1	48.1457	48.1582	-0.0125
20	4	2	0	49.5133	49.4921	0.0213
24	4	2	2	54.6214	54.5871	0.0343
27	3	3	3	58.1733	58.2039	-0.0306

The final (best) R_{Bragg} value was 9.7 %, calculated from the part of the pattern close to the calculated peaks. Including all peaks gives slightly higher residual values. As can be seen from the Rietveld profile (Figure S2), several weak peaks are present in the pattern, except for those derived from the cubic unit cell. These are most likely related to witherite, or similar structures but this conclusion cannot be easily reached. The arrangement of carbonate ions around radium(II) in radium carbonate and its electron density map are shown in Figures S3 and S4, respectively.

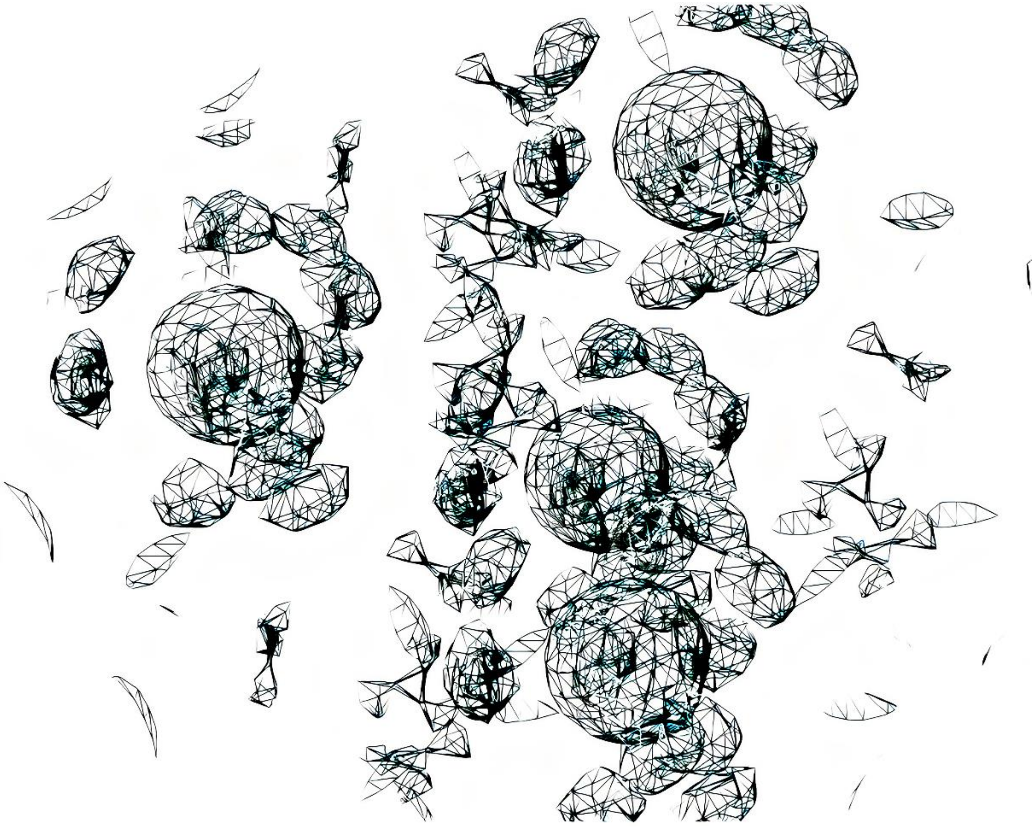

Figure S3. Electron density map of Ra(Ba)CO_3 obtained from FOX and the Monte Carlo direct space structure solution

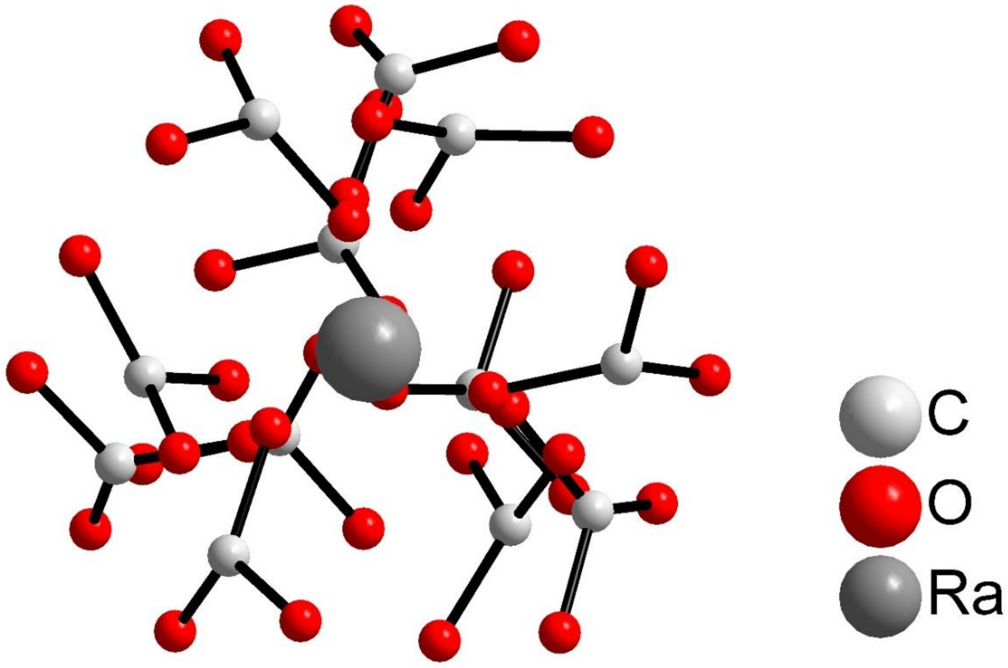

Figure S4. The disordered arrangement of carbonate ions around radium(II) in radium carbonate

References

1. Davies, C. W.; Shedlovsky, T., Ion association. *Journal of The Electrochemical Society* **1964,** *111* (3), 85C-86C https://doi.org/10.1149/1.2426129
2. Brønsted, J. N., Studies on solubility. IV. The principle of the specific interaction of ions. *Journal of the American Chemical Society* **1922,** *44* (5), 877-898 http://dx.doi.org/10.1021/ja01426a001
3. Brønsted, J. N., Calculation of the osmotic and activity functions in solutions of uni-univalent salts. *Journal of the American Chemical Society* **1922,** *44* (5), 938-948 http://dx.doi.org/10.1021/ja01426a003
4. Scatchard, G., Concentrated solutions of strong electrolytes. *Chemical Reviews* **1936,** *19* (3), 309-327 http://dx.doi.org/10.1021/cr60064a008
5. Guggenheim, E.; Turgeon, J., Specific interaction of ions. *Transactions of the Faraday Society* **1955,** *51*, 747-761
6. Ribeiro, A. C.; Lobo, V. M.; Burrows, H. D.; Valente, A. J.; Sobral, A. J.; Amado, A. M.; Santos, C. I.; Esteso, M. A., Mean distance of closest approach of potassium, cesium and rubidium ions in aqueous solutions: Experimental and theoretical calculations. *Journal of Molecular Liquids* **2009,** *146* (3), 69-73 https://doi.org/10.1016/j.molliq.2009.02.006
7. Ciavatta, L., The specific interaction theory in equilibrium analysis: some empirical rules for estimating interaction coefficients of metal-ion complexes. *Annali di Chimica* **1990,** *80* (5-6), 255-263
8. Bjerrum, N. Studies on basic chromic compounds. A contribution to the theory of hydrolysis. Dissertation, Copenhagen, 1908.
9. Bjerrum, N., K. Dan. Vidensk. Selsk. Mat. Fys. Medd. **1926,** (7), 1-48
10. Spahiu, K.; Puigdomenech, I., On weak complex formation: re-interpretation of literature data on the Np and Pu nitrate complexation. *Radiochimica Acta* **1998,** *82* (s1), 413-420 https://doi.org/10.1524/ract.1998.82.special-issue.413
11. Busenberg, E.; Plummer, L. N., The solubility of $BaCO_3$(cr) (witherite) in CO_2-H_2O solutions between 0 and 90°C, evaluation of the association constants of $BaHCO_3^+$(aq) and $BaCO_3^0$(aq) between 5 and 80°C, and a preliminary evaluation of the thermodynamic properties of Ba^{2+}(aq). *Geochimica et Cosmochimica Acta* **1986,** *50* (10), 2225-2233 https://doi.org/10.1016/0016-7037(86)90077-3
12. Busenberg, E.; Plummer, L. N.; Parker, V. B., The solubility of strontianite ($SrCO_3$) in CO_2-H_2O solutions between 2 and 91°C, the association constants of $SrHCO_3^+$(aq) and $SrCO_3^0$(aq) between 5 and 80°C, and an evaluation of the thermodynamic properties of Sr^{2+}(aq) and $SrCO_3$(cr) at 25°C and 1 atm total pressure. *Geochimica et Cosmochimica Acta* **1984,** *48* (10), 2021-2035 https://doi.org/10.1016/0016-7037(84)90383-1
13. Monnin, C., A thermodynamic model for the solubility of barite and celestite in electrolyte solutions and seawater to 200°C and to 1 kbar. *Chemical Geology* **1999,** *153* (1–4), 187-209 http://dx.doi.org/10.1016/S0009-2541(98)00171-5
14. Matyskin, A. V.; Brown, P. L.; Ekberg, C., Weak barium and radium hydrolysis using an ion exchange method and its uncertainty assessment. *The Journal of Chemical Thermodynamics* **2019,** *128*, 362-371 https://doi.org/10.1016/j.jct.2018.08.037
15. Butler, J. N.; Huston, R., Activity coefficients and ion pairs in the systems sodium chloride-sodium bicarbonate-water and sodium chloride-sodium carbonate-water. *The Journal of Physical Chemistry* **1970,** *74* (15), 2976-2983 https://doi.org/10.1021/j100709a024
16. Capewell, S.; Buchner, R.; Hefter, G.; May, P., Dielectric relaxation of aqueous Na_2CO_3 solutions. *Physical Chemistry Chemical Physics* **1999,** *1* (8), 1933-1937 https://doi.org/10.1039/A900812H

17. Capewell, S. G.; Hefter, G.; May, P. M., Potentiometric Investigation of the Weak Association of Sodium and Carbonate Ions at 25°C. *Journal of Solution Chemistry* **1998,** *27* (10), 865-877 https://doi.org/10.1023/A:1022696726873
18. Crea, F.; Stefano, C. D.; Gianguzza, A.; Piazzese, D.; Sammartano, S., Protonation of carbonate in aqueous tetraalkylammonium salts at 25°C. *Talanta* **2006,** *68* (4), 1102-1112 https://doi.org/10.1016/j.talanta.2005.07.025
19. Daniele, P. G.; Foti, C.; Gianguzza, A.; Prenesti, E.; Sammartano, S., Weak alkali and alkaline earth metal complexes of low molecular weight ligands in aqueous solution. *Coordination Chemistry Reviews* **2008,** *252* (10), 1093-1107 https://doi.org/10.1016/j.ccr.2007.08.005
20. Garrels, R. M.; Thompson, M. E., A chemical model for sea water at 25 degrees C and one atmosphere total pressure. *American Journal of Science* **1962,** *260* (1), 57-66 https://doi.org/10.2475/ajs.260.1.57
21. Garrels, R. M.; Thompson, M. E.; Siever, R., Control of carbonate solubility by carbonate complexes. *American Journal of Science* **1961,** *259* (1), 24-45 https://doi.org/10.2475/ajs.259.1.24
22. Mojica Prieto, F. J.; Millero, F. J., The values of $pK_1 + pK_2$ for the dissociation of carbonic acid in seawater. *Geochimica et Cosmochimica Acta* **2002,** *66* (14), 2529-2540 https://doi.org/10.1016/S0016-7037(02)00855-4
23. Pytkowicz, R. M.; Hawley, J. E., Bicarbonate and carbonate ion-pairs and a model of seawater at 25°C. *Limnology and Oceanography* **1974,** *19* (2), 223-234 https://doi.org/10.4319/lo.1974.19.2.0223
24. Schmidt, C., Raman spectroscopic determination of carbon speciation and quartz solubility in $H_2O+Na_2CO_3$ and $H_2O+NaHCO_3$ fluids to 600 °C and 1.53 GPa. *Geochimica et Cosmochimica Acta* **2014,** *145*, 281-296 https://doi.org/10.1016/j.gca.2014.09.009
25. Sipos, P.; Bolden, L.; Hefter, G.; May, P. M., Raman Spectroscopic Study of Ion Pairing of Alkali Metal Ions with Carbonate and Sulfate in Aqueous Solutions. *Australian Journal of Chemistry* **2001,** *53* (10), 887-890 https://doi.org/10.1071/CH00126
26. Stefánsson, A.; Bénézeth, P.; Schott, J., Carbonic acid ionization and the stability of sodium bicarbonate and carbonate ion pairs to 200°C – A potentiometric and spectrophotometric study. *Geochimica et Cosmochimica Acta* **2013,** *120*, 600-611 https://doi.org/10.1016/j.gca.2013.04.023
27. Wu, J.; Wang, S.; Zheng, H., The influence of ionic strength on carbonate-based spectroscopic barometry for aqueous fluids: an in-situ Raman study on Na_2CO_3-NaCl solutions. *Scientific Reports* **2016,** *6*, 39088 https://doi.org/10.1038/srep39088
